# The Impact of Noncavity-Distorting Intramural Fibroids on the Efficacy of In Vitro Fertilization-Embryo Transfer: An Updated Meta-Analysis

**DOI:** 10.1155/2018/8924703

**Published:** 2018-09-04

**Authors:** Xiaodan Wang, Li Chen, Hengyu Wang, Qin Li, Xiru Liu, Hongbo Qi

**Affiliations:** ^1^The Department of Obstetrics and Gynecology, The First Affiliated Hospital of Chongqing Medical University, Chongqing 400016, China; ^2^State Key Laboratory of Maternal and Fetal Medicine of Chongqing Municipality, Chongqing Medical University, Chongqing 400016, China; ^3^International Collaborative Laboratory of Reproduction and Development of Chinese Ministry of Education, Chongqing Medical University, Chongqing 400016, China

## Abstract

**Aim:**

To address the impact of noncavity-distorting intramural fibroids on the efficacy of in vitro fertilization-embryo transfer (IVF-ET) outcomes.

**Methods:**

The PubMed, Web of Science, Embase, Cochrane Library, and China National Knowledge Infrastructure were searched systematically. A meta-analysis was performed based on comparative or cohort studies that explored the impact of noncavity-distorting intramural fibroids on the efficacy of IVF-ET treatment. The IVE-ET outcomes of study group (women with noncavity-distorting intramural fibroids) and control group (women without fibroids) were compared, including live birth rate (LBR), clinical pregnancy rate (cPR), implantation rate (IR) , miscarriage rate (MR), and ectopic pregnancy rate (ePR).

**Results:**

A total of 28 studies involving 9189 IVF cycles were included. Our meta-analysis showed a significant reduction of LBR in the study group compared to control group (RR = 0.82, 95% CI: 0.73-0.92, and P = 0.005). In addition, it indicated that study group had a significant reduction in cPR (RR = 0.86, 95% CI: 0.80-0.93, P = 0.0001) and IR (RR = 0.90, 95% CI: 0813-1.00, P = 0.04) and have a significantly increase in MR (RR = 1.27, 95% CI: 1.08-1.50, and P = 0.004) compared with control group.

**Conclusions:**

The present evidence suggests that noncavity-distorting intramural fibroids would significantly reduce the IR, cRP, and LBR and significantly increase the MR after IVF treatment, but it would not significantly increase the ePR.

## 1. Introduction

The uterine fibroids are the most common benign tumor in women, especially during their reproductive years [1–3]. An estimated 70-80% of women will have a fibroid in their lifetime [3]. Since there is a variation in number, size, and location of the fibroids, the clinical effects of uterine fibroids are heterogeneous, including pelvic pain, menorrhagia, impingement, infertility, spontaneous abortion, pelvic outlet obstruction, preterm delivery, and related complications [4–6]. Consensus showed that uterine fibroids, especially submucosal fibroids, may have adverse impacts on fertility of women by anatomically distorting the endometrial cavity and altering the intracavitary environment [6–8]. Several studies indicated that the cavity-involved fibroids may cause worse outcomes of in vitro fertilization-embryo transfer (IVF-ET) [7, 9].

However, many intramural fibroids have no endometrial cavity distortion, of which the effect on the outcomes of IVF-ET remains unclear. The management of this kind of fibroids is controversial. It is quite remarkable that there is 12.6% infertility women undergoing IVF complicated with uterine fibroids [10]. Considering the extension of reproductive timeline of women due to the improvement of medical care and the current trend of childbearing postponement, the proportion of infertility issues owing to uterine fibroids may sustain a relatively high rate of growth. It seems important to figure out whether the noncavity-distorting intramural fibroids will impact the efficacy of IVF-ET treatment and the suitable management of this kind of fibroids in women with infertility issues. Once the impacts are clear, this will provide instructions on the management of the noncavity-distorting intramural fibroids.

## 2. Methods

### 2.1. Search Question

Do noncavity-distorting intramural fibroids have impacts on the in vitro fertilization-embryo transfer (IVF-ET) outcomes?

### 2.2. Search Strategy

The keywords included uterine fibroids (“fibroids”, “leiomyomas”, and “myomas”) and in vitro fertilization (“in vitro fertilization”, “fertilization-in vitro”, “assisted reproductive technology”, “intracytoplasmic sperm injection”, “sperm injection intracytoplasmic”, “reproductive techniques assisted”, “embryo transfer”, and “embryo implantation”). PubMed was systematically searched primarily, and the Web of Science, Embase, Cochrane Library, and China National Knowledge Infrastructure (CNKI) were searched for supplementary. The deadline for searching was April 10, 2018. All unduplicated records were contained in the literature pool for screening, and there are no language restrictions here.

### 2.3. Including Criteria

The studies were eligible if they satisfied the following criteria: (1) the target population was the infertility women who underwent IVF-ET and had control group; (2) the exposure of the study group was the existence of noncavity-distorting intramural fibroids and no fibroids in the control group; (3) the outcomes of interest included the LBR, cPR, IR, MR, and/or ePR. Studies containing women with intramural fibroids protruding into the endometrial cavity were excluded, as well as women with submucosal fibroids.

### 2.4. Study Selection and Data Extraction

The studies selection was performed by two reviewers independently. First, the titles and abstracts were scrutinized to identify studies which were likely to meet the prespecified criteria and all full texts of those studies were obtained. Second, the full texts were viewed carefully to obtain studies that meet the predefined inclusive criteria. The references of those articles were also examined to identify potential studies that were not captured by our databases searching. The repetitive articles are excluded. Any divergences about studies selection were resolved by consensus of all researchers of this study. The following information was collected if available, including type of study, the selection criteria, group size, fibroids status, demographic characteristics, and IVF-ET outcomes.

### 2.5. Quality Assessment

The Newcastle-Ottawa Scale (NOS) [11] was used to evaluate the quality of nonrandomized controlled studies. The score of NOS ranged from 0 to 9. Studies with scores ≥ 7 were regarded to have a low risk of bias; studies with scores of 4–6 were regarded to have a moderate risk of bias; and studies with scores < 4 were regarded to have a high risk of bias [11]. The publication bias was evaluated by funnel plots and tested by Begg's test, which were performed in the Stata 14.0 (Stata Corp, College Station, Texas, USA).

### 2.6. Outcome Measures and Statistical Analysis

The primary outcome was LBR, and the secondary outcomes included cPR, IR, MR, and ePR. The heterogeneity was evaluated by Q test and was presented with I^2^ and P values [12]. When the P > 0.1 and I^2^ ≤ 50%, the heterogeneity was regarded to be nonsignificant and then the fixed effect model was applied in meta-analysis. Otherwise, the heterogeneity was regarded to be significant and then influence analysis was performed to find out the origin of the heterogeneity. If the influence analysis failed to find out the origin of heterogeneity, the random effects model was employed. Heterogeneity test and meta-analysis was performed by the Review Manager 5.3 software (Cochrane Collaboration, Oxford, UK). The statistical differences were presented by risk ratio (RR) and were tested by the Z test. If the P was less than 0.05, the difference of the indicators was statistically significant.

## 3. Result

**Figure 1:**
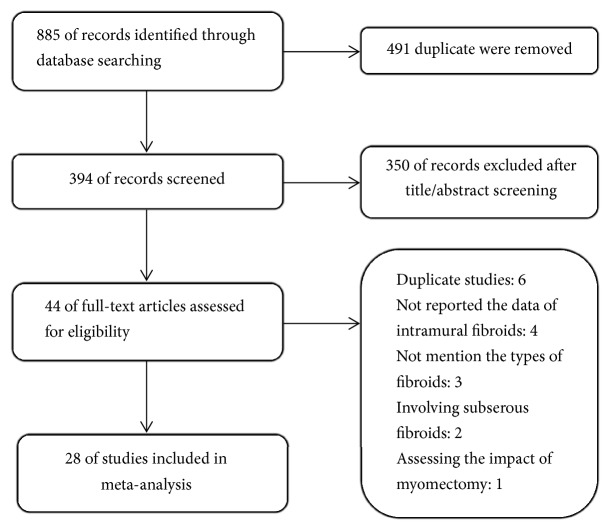
Flow diagram of studies selection.

In total, 885 records were retrieved from the prementioned databases, 491 of which were duplicate records and were subsequently rejected (Figure 1). Furthermore, 350 were removed after title and abstract screening. All the full articles of the 44 remaining studies were obtained and evaluated. Additional 16 studies were excluded. Six of these studies used the same data involved in other included studies [13–18], and four failed to report the data of the women with noncavity-distorting intramural fibroids individually [2, 19–21]; three did not mention the types of the fibroid [22–24]; two contained women with predominantly subserous fibroids [10, 25]; one assessed the impact of myomectomy of fibroids before IVF-ET [26]. At last, 28 studies were included in this meta-analysis [6, 27–53] (Table 1). All the 19 studies in the precedent meta-analysis of Sunkara et al. were identified by this searching strategy [54].

### 3.1. Assessment of the Included Studies

All the eligible studies comprising 9189 IVF cycles were controlled studies, of which 7 were designed as prospective studies involving 1534 IVF cycles. The quality of all included studies was evaluated item by item rigorously according to the NOS of cohort study [11]. Each of them has a NOS score greater than 6, which indicates that these studies have a low risk of relevant bias (Table 2). Especially, 23 of these studies have controlled the potential confounding factors by matching the age, number of embryos, number of cycles, and/or other factors between the study and the control groups. 5 other studies randomly had women enrolled into the control group from the same population without factors matched [28, 30, 32, 44, 51], but in the studies of Lu and Long, the age and some other important factors had no statistical difference between the study and control groups [28, 30].

### 3.2. Live Birth Rate

**Figure 2:**
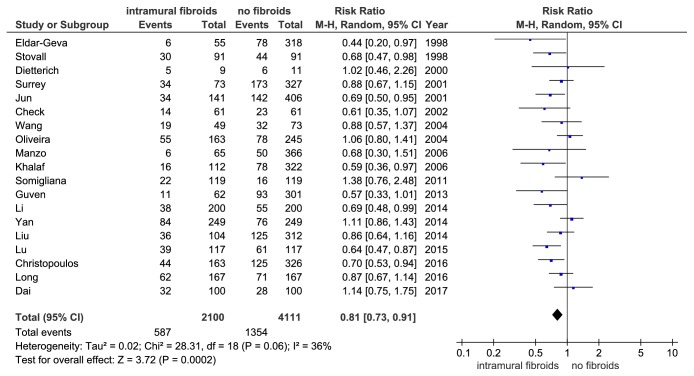
The effect of noncavity-distorting intramural fibroids on live birth rate (LBR) after IVF-ET.

Nineteen eligible studies comprising 6211 IVF cycles presented data on LBR. Meta-analysis of these studies showed that women with noncavity-distorting intramural fibroids had a significant reduction in LBR compared to women without fibroids (RR = 0.81, 95% CI: 0.73-0.91, P = 0.0002, and Figure 2). Begg's test showed no significant publication bias (P = 0.234). The I^2^ value was 36% (P = 0.06) indicating a little variability among these studies, but the influence analysis did not find any studies that dominantly contributed to the heterogeneity. So the random effects model was employed.

**Figure 3:**
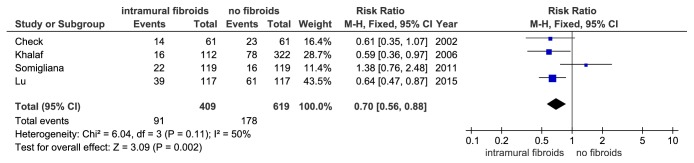
The effect of noncavity-distorting intramural fibroids on live birth rate (LBR) after IVF-ET from prospective studies.

**Figure 4:**
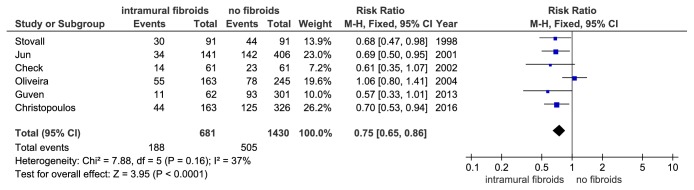
The effect of noncavity-distorting intramural fibroids on live birth rate (LBR) after IVF-ET in women undergoing their first IVF cycle.

Pooled analysis of the four prospective studies that reported LBR indicates a significantly lower LBR in study group compared to the control group (RR = 0.70, 95%CI: 0.56-0.88, P = 0.002, and Figure 3). The I^2^ values were 50% (P = 0.11) indicating no significant heterogeneity. Six of the nineteen studies only involving women who underwent their first IVF cycle also indicated a considerable 25% reduction in the study group compared to the control group in LBR (RR = 0.75, 95% CI: 0.65-0.86,P = 0.0001, and Figure 4). The I^2^ values were 37% (P = 0.16) indicating little variability.

### 3.3. Clinical Pregnancy Rate

**Figure 5:**
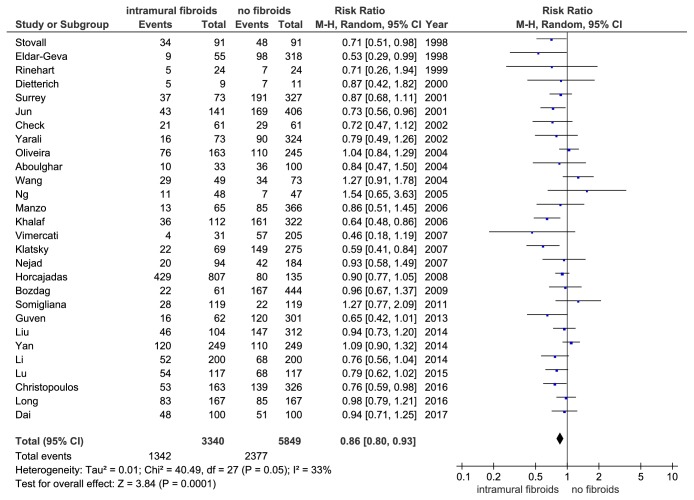
The effect of noncavity-distorting intramural fibroids on clinical pregnancy rate (cPR) after IVF-ET.

The data about cPR were extracted from all the 28 studies involving 9189 IVF cycles. There was no significant publication bias (P = 0.186) confirmed by Begg's test. There was a little heterogeneity (P = 0.05, I^2^ =33%) among the included studies but the influence analysis failed to find any studies obviously responsible for the heterogeneity. Subsequently, a random effect model was chosen. The results showed a significantly lower cPR in women with noncavity-distorting intramural fibroids than that in the women without fibroids (RR = 0.86, 95% CI: 0.80-0.93, P = 0.0001, and Figure 5).

**Figure 6:**
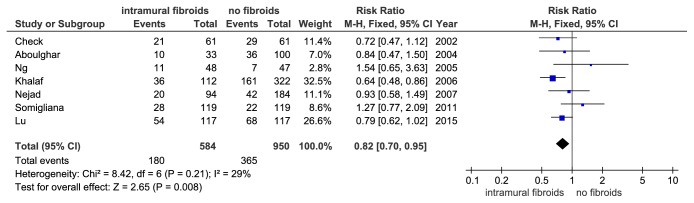
The effect of noncavity-distorting intramural fibroids on clinical pregnancy rate (cPR) after IVF-ET from prospective studies.

**Figure 7:**
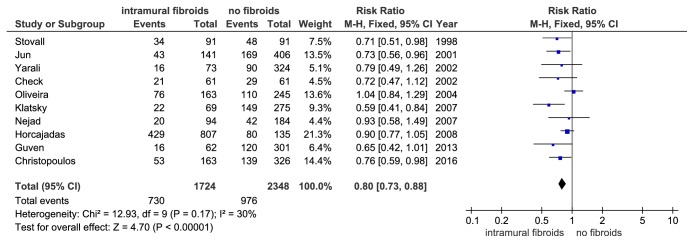
The effect of noncavity-distorting intramural fibroids on clinical pregnancy rate (cPR) after IVF-ET in women undergoing their first IVF-ET cycle.

Of these, there were 7 prospective studies. And the I^2^ values were 29% (P = 0.210) indicating no significant inconsistence among these studies. The meta-analysis of these prospective studies indicated that the study group had a significant reduction in cPR. (RR = 0.82, 95%CI: 0.70–0.95, P = 0.008, and Figure 6). Besides, meta-analysis of the 10 studies, only containing women who underwent their first IVF treatment cycle, obtained a similar result (RR = 0.80, 95% CI: 0.73–0.88, P < 0.00001, and Figure 7). There was no definite evidence for significant inconsistency among these studies (I^2^ = 30%, P = 0.17).

### 3.4. Implantation Rate

**Figure 8:**
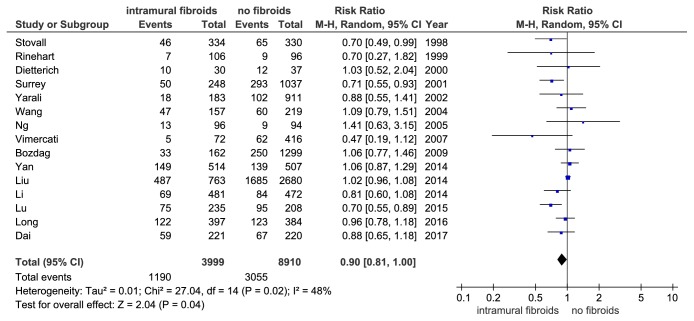
The effect of noncavity-distorting intramural fibroids on implantation rate (IR) after IVF-ET.

For the analysis of IR, pooled analysis of the fifteen included studies that displayed a lower IR in women with noncavity-distorting intramural fibroids than in women without fibroids (RR = 0.90, 95% CI: 0.81-1.00, P = 0.04, and Figure 8). Begg's test showed that the publication bias was not significant (P = 0.843). A random effects model was applied because of the heterogeneity of the IR (I^2^ = 48%, P = 0.02) among the included studies.

### 3.5. Miscarriage Rate

**Figure 9:**
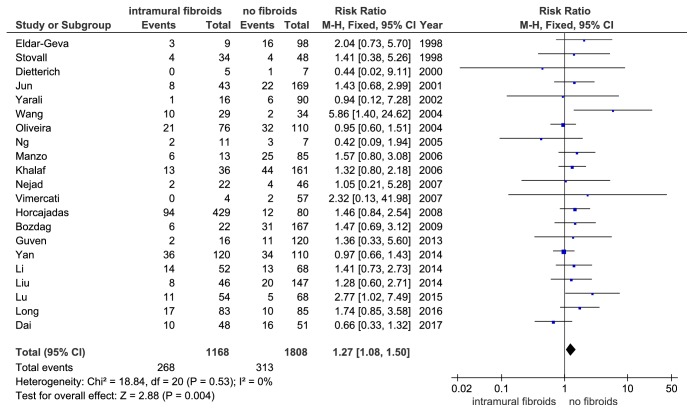
The effect of noncavity-distorting intramural fibroids on miscarriage rate (IR) after IVF-ET.

A total of 21 of the 28 studies with IVF cycles reported the MR or abortion rate as an outcome. Begg's test showed no existence of significant publication bias (P = 0.976). The I^2^ value was 0% (P = 0.53) and indicated no heterogeneity among these studies; therefore the fixed effect model was employed. Meta-analysis of MR in the included studies showed that women with noncavity-distorting intramural fibroids had a significantly increase in miscarriage/abortion rate compared with women without fibroids (RR =1.27, 95% CI: 1.08-1.50, P = 0.004, and Figure 9).

### 3.6. Ectopic Pregnancy Rate

**Figure 10:**
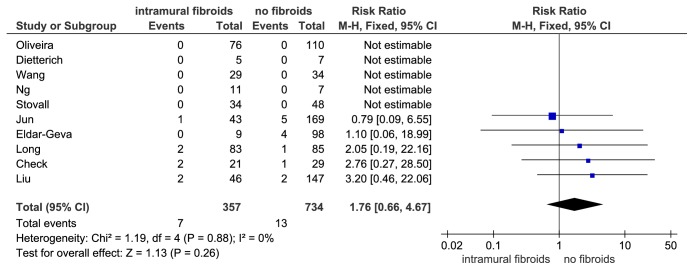
The effect of noncavity-distorting intramural fibroids on ectopic pregnancy rate (ePR) after IVF-ET.

Ten studies with 1091 IVF cycles reported the ectopic pregnancy rate (ePR) as an outcome. However, 5 of the 10 studies showed no ectopic pregnancy in both study group and control group [43–45, 51, 53], which were not fit for meta-analysis. Begg's test showed there was no existence of significant publication bias among the included studies (P = 0.462). The I2 value was 0% (P = 0.88) indicating no heterogeneity among these studies, so the fixed effect model was employed. Results of the meta-analysis on ePR showed that women with noncavity-distorting intramural fibroids had no significant increase in ePR compared with women without fibroids (RR = 1.76, 95% CI: 0.66-4.67, P = 0.260, and Figure 10).

## 4. Discussion

Intramural fibroids without involving uterine cavity remain a clinical disturbance in women with fertility difficulty. It is not clear whether this kind of fibroids would impact the IVF-ET outcomes. If so, fibroids removal before IVF treatment may improve the IVF-ET outcomes. Jun et al. compared retrospectively 141 women with noncavity-distorting fibroids with 406 women without fibroids undergoing their first IVF cycle and concluded that the noncavity-distorting intramural fibroids have no impact on IVF-ET outcomes [50]. On the contrary, Eldar-Geva et al. compared the outcome of IVF-ET between 88 women with the noncavity-distorting fibroids and 318 women without fibroids, and this revealed considerable reduction in implantation rate (IR) (6.4% vs 15.8%, P<0.05) and clinical pregnant rate (cPR) (16.4% vs 30.1%, P<0.005) in women with noncavity-distorting intramural fibroids [6]. In addition, several controlled trails found that the LBR and cPR were significantly decreased in women undergoing IVF-ET treatment with noncavity-distorting intramural fibroids compared with those without fibroids [37, 42, 48, 53].

Eight years ago, Sunkara et al. enrolled 19 relevant studies before 2009 and conducted a similar meta-analysis and concluded that the noncavity-distorting intramural fibroids had adverse impact on IVF-ET [54]. And, Metwally et al. presented another meta-analysis containing 10 studies and concluded that there is no evidence of a significant effect for the noncavity-distorting intramural fibroids on clinical pregnancy rate, live birth rate, or miscarriage rate after IVF-ET [55]. Our meta-analysis included a series of new relevant studies and explored the potential effects of intramural fibroids without endometrial cavity distortion on IVF-ET outcomes. In this meta-analysis, a total of 28 studies involving 9189 IVF cycles were included. Our meta-analysis showed a significant reduction of LBR in the study group compared to control group (RR = 0.82, 95% CI: 0.73-0.92, and P = 0.005). In addition, it indicated that study group had a significant reduction in cPR (RR = 0.86, 95% CI: 0.80-0.93, and P = 0.0001) and IR (RR = 0.90, 95% CI: 0813-1.00, and P = 0.04) and have a significantly increase in MR (RR = 1.27, 95% CI: 1.08-1.50, and P = 0.004) compared with control group. This implies that the noncavity-distorting intramural fibroids will impact the efficacy of IVF-ET indeed.

The mechanism has not been well established for the adverse impacts of noncavity-distorting intramural fibroids to the efficacy of IVF treatment. Some precedent studies and reviews showed that the noncavity-distorting intramural fibroids probably change the uterine vascular supply, myometrial contraction-relaxation, and endometrial function [43, 54]. Sunkara et al. for the first time conducted a meta-analysis on this subject [54]. Their results showed that women with noncavity-distorting intramural fibroids have a considerable reduction in LBR and cPR but have no significant reduction in IR or significant increase in MR compared with women without fibroids. These may indicate that the adverse impacts of noncavity-distorting intramural fibroids on the efficacy of IVF-ET treatment are primarily taken on the course of pregnancy. However, enrolling additional 9 studies, our meta-analysis found that the presence of noncavity-distorting intramural fibroids significantly reduces the IR, the cPR, and the LBR by 6%, 14%, and 19%, respectively, compared with women without fibroids. On the other hand, MR was significantly increased by 27%. These results probably indicate that the adverse effects of noncavity-distorting intramural fibroids probably persist on the proceedings from embryo implantation and pregnancy to childbirth. Except for these, noncavity-distorting intramural fibroids may not affect the occurrence of ectopic pregnancy.

The included studies were obtained from systematic literatures search and extracted carefully by two reviewers independently. The NOS was applied to evaluate the quality of nonrandomized controlled studies. Studies with NOS score less than 6 would be excluded, but all the included studies of this meta-analysis have a NOS score no less than 7. The publication bias was assessed by funnel plot analyses and tested by Begg's test. All the funnel plots were symmetrical by inspection and the relevant P values of Begg's test were greater than 0.05, indicating that publication biases of the including studies were unlikely.

The deficiency of this meta-analysis is mainly due to the heterogeneity among the included studies. Firstly, 7 of the 28 studies were prospective [30, 35, 39, 42, 43, 46, 48], but the others were retrospective in design. Secondly, 10 of the 28 studies recorded a woman once because they only enrolled the first cycle of a woman undergoing IVF treatment [29, 34, 37, 39, 40, 45, 47, 48, 50, 53], but the others may have recorded a woman more than one time because they enrolled every cycle of the previous IVF treatments. To validate these potential origins of heterogeneity, we performed some subgroup analyses. Both the meta-analyses of prospective studies and the studies only involving women who underwent their first IVF-ET cycle showed consistent results with the overall analysis in the LBR and cPR. For the IR, neither meta-analysis of prospective studies nor the studies with only women undergoing their first IVF-ET cycle showed significantly different outcome between women with noncavity-distorting intramural fibroids and women without fibroids. These nonsignificant results may be limited by the much smaller number of the included studies than that of overall analysis. Besides, the number, mean size of fibroids, and age of enrolled women were also various among the studies. Nevertheless, the variances seemed to be limited, because the mean age varied between the ages of 33 and 43, the number of fibroids varied between 1 and 8, and the mean size of fibroids varied between 15 and 50 micrometers.

On the whole, it is undeniable that these included nonrandomized control studies had some potential bias, and the heterogeneity of these studies indeed exists in this meta-analysis, but there are no more prospective randomized control trails up to now, and the heterogeneity was addressed as much as possible in this meta-analysis. Therefore, this systemic review and meta-analysis may represent the most comprehensive and reliable evidence. Despite the adverse impacts on IVF outcomes of noncavity-distorting intramural fibroids, there is still no definite evidence suggesting routine myomectomy for this kind of fibroids. Therefore, there is a need for well-designed randomized control trial to explore if the myomectomy for noncavity-distorting intramural fibroids would improve the efficacy of IVF-ET treatment or not.

## Figures and Tables

**Table 1:** Characteristics of the studies included in this review (R: reproductive; P: perspective; IR: implantation rate; cPR: clinical pregnancy rate; LBR: live birth rate; MR: miscarriage rate; ePR: ectopic pregnancy rate).

Reference	Type		Study group								Control group						
		No. of IVF cycles	Age (year)	Size of fibroids (mm)	No. of fibroids	IR	cPR	LBR	MR	ePR	No. of IVF cycles	Age	IR	cPR	LBR	MR	ePR
Eldar-Geva [6] 1998	R	55	35.3±1.2	23.7±7.1	1.82±0.35	6.4%	9/55	6/55	3/9	0/9	318	35.5±0.4	15.8%	98/318	78/318	16/98	4/98
Stovall [53] 1998	R	91	35.8±4.1	8-50	1.8±0.8	46/334	34/91	30/91	4/34	0/34	91	35.9±3.4	65/330	48/91	44/91	4/48	0/48
Rinehart [52] 1999	R	24	-	5-32.5	1-5	7/106	5/24	-	-	-	24	-	9/96	7/24	-	-	-
Dietterich [51] 2000	R	9	35+	6-26	1-6	10/30	5/9	5/9	0/5	0/5	11	35	12/37	7/11	6/11	1/7	0/7
Jun [50] 2001	R	141	36.9±4.0	19.3±12.6	-	-	43/141	34/141	8/43	1/43	406	34.8±4.2	-	169/406	142/406	22/169	5/169
Surrey [49] 2001	R	73	31-45	-	-	50/248	37/73	34/73	-	-	327	-	293/1037	191/327	173/327	-	-
Check [48] 2002	P	61	36.6±4.5	15±0.9	2.1±0.18	-	21/61	14/61	-	2/21	61	36.6±4.5	-	29/61	23/61	-	1/29
Yarali [47] 2002	R	73	35.9±3.3	30±18	3.1±2.0	18/183	16/73	-	1/16	-	324	35.6±3.9	102/911	90/324	-	6/90	-
Aboulgha [46] 2004	P	33	35.9±3.1	-	-	-	10/33	-	-	-	100	36.4±2.3	-	36/100	-	-	-
Oliveira [45] 2004	R	163	35.1±3.6	4-69	1-4	-	76/163	55/163	21/76	0/76	245	35.1±3.6	-	110/245	78/245	32/110	0/110
Wang [44] 2004	R	49	43±5.1	<30.3	-	47/157	29/49	19/49	10/29	0/29	73	39.5±5.7	60/219	34/73	32/73	2/34	0/34
Ng [43] 2005	P	48	27-40	-3	1-6	13/96	11/48	-	2/11	0/11	47	27-4	9/94	7/47	4/47	3/7	0/7
Khalaf [42] 2006	P	112	36.4±3.9	23±11	1.8 ± 0.8	-	36/112	16/112	13/36	-	322	34.6±3.9	-	161/322	78/322	44/161	-
Manzo [41] 2006	R	65	<37	20-50	-	-	13/65	6/65	6/13	-	366	<37	-	85/366	50/366	25/85	-
Klatsky [40] 2007	R	69	42.7±0.5	28	-	-	22/69	-	-	-	275	40.9±0.28	-	149/275	-	-	-
Nejad [39] 2007	P	94	33.9±3.4	-	-	-	20/94	-	2/22	-	184	33.3 ±3.6	-	42/184	-	4/46	-
Vimercati [38] 2007	R	31	34.8±5.3	-	-	5/72	4/31	-	0/4	-	205	33.8±4.5	62/416	57/205	-	2/57	-
Horcajadas [37] 2008	R	807	-	-	-	-	429/807	-	94/429	-	135	-	-	80/135	-	12/80	-
Bozdag [36] 2009	R	61	35.3±4.5	-	-	33/162	22/61	-	6/22	-	444	34.5 ± 3.6	250/1299	167/444	-	31/167	-
Somigliana [35] 2011	P	119	37.6±3.0	22.0±10.0	1-5	-	28/119	22/119	-	-	119	37.6±3.0	-	22/119	16/119	-	-
Guven [34] 2013	R	62	33.0±4.0	49.6±12.3	1	-	16/62	11/62	2/16	-	301	32.66±5.3	-	120/301	93/301	11/120	-
Yan [31] 2014	R	249	35.0±4.0	<60	-	149/514	120/249	84/249	36/120	-	249	35.0±4.0	139/507	110/249	76/249	34/110	-
Li [33] 2014	R	200	-	-	-	69/481	52/200	38/200	14/52	-	200	-	84/472	68/200	55/200	13/68	-
Liu [32] 2014	R	104	34.1±3.9	24.2±13.7	-	687/763	46/104	36/104	8/46	2/46	312	30.7± 4.2	1685/2680	147/312	125/312	20/147	2/147
Lu [30] 2015	P	117	33.0	<40	-	75/235	54/117	39/117	11/54	-	117	33.0	95/208	68/117	61/117	5/68	-
Christopoulos [29] 2016	R	163	35.7±3.6	25	1-8	-	53/163	44/163	-	-	326	35.7±3.6	-	139/326	125/326	-	-
Long [28] 2016	R	167	35.9± 3.5	-	-	122 /397	83 /167	62/167	17 /83	2 /83	167	35.9±3.5	123 /384	85 /167	71/167	10 /85	1/85
Dai [27] 2017	R	100	36.4±3.9	-	-	59 /221	48 /100	32/100	10 /48	-	100	36.2 ± 4.3	67 /220	51 /100	28/100	16 /51	-

**Table 2:** Appraisal of methodological quality (Newcastle-Ottawa Scale).

**Reference**	**Case-cohort representative**	**Selection of non- exposed control**	**Ascertainment of exposure**	**Outcome negative at start**	**Comparability by design**	**Comparability by analysis**	**Outcome assessment**	**Duration of follow-up**	**Adequacy of follow-up**	**Score**
Eldar-Geva [6] 1998	√	√	√	√	√	×	√	√	√	8
Stovall [53] 1998	√	√	√	√	√	×	√	√	√	8
Rinehart [52] 1999	√	√	√	√	√	×	√	√	√	8
Dietterich [51] 2000	√	√	√	√	×	×	√	√	√	7
Jun [50] 2001	√	√	√	√	×	√	√	√	√	8
Surrey [49] 2001	√	√	√	√	√	√	√	√	√	9
Check [48] 2002	√	√	√	√	√	×	√	√	√	8
Yarali [47] 2002	√	√	√	√	√	×	√	√	√	8
Aboulgha [46] 2004	√	√	√	√	√	×	√	√	√	8
Oliveira [45] 2004	√	√	√	√	√	×	√	√	√	8
Wang [44] 2004	√	√	√	√	×	×	√	√	√	7
Ng [43] 2005	√	√	√	√	√	×	√	√	√	8
Khalaf [42] 2006	√	√	√	√	×	√	√	√	√	8
Manzo [41] 2006	√	√	√	√	×	√	√	√	√	8
Klatsky [40] 2007	√	√	√	√	×	√	√	√	√	8
Nejad [39] 2007	√	√	√	√	√	×	√	√	√	8
Vimercati [38] 2007	√	√	√	√	×	√	√	√	√	8
Horcajadas [37] 2008	√	√	√	√	×	√	√	√	√	8
Bozdag [36] 2009	√	√	√	√	√	×	√	√	√	8
Somigliana [35] 2011	√	√	√	√	√	×	√	√	√	8
Guven [34] 2013	√	√	√	√	√	×	√	√	√	8
Yan [31] 2014	√	√	√	√	√	×	√	√	√	8
Li [33] 2014	√	√	√	√	√	×	√	√	√	8
Liu [32] 2014	√	√	√	√	×	×	√	√	√	7
Lu [30] 2015	√	√	√	√	×	√	√	√	√	8
Christopoulos [29] 2016	√	√	√	√	√	×	√	√	√	8
Long [28] 2016	√	√	√	√	×	√	√	√	√	8
Dai [27] 2017	√	√	√	√	√	×	√	√	√	8

## Data Availability

The data used to support the findings of this study are included within the article.
